# Giant Uniaxial Magnetocrystalline Anisotropy in SmCrGe_3_

**DOI:** 10.1021/jacs.4c10056

**Published:** 2024-10-28

**Authors:** Mingyu Xu, Yongbin Lee, Xianglin Ke, Min-Chul Kang, Matt Boswell, Sergey L. Bud’ko, Lin Zhou, Liqin Ke, Mingda Li, Paul C. Canfield, Weiwei Xie

**Affiliations:** † Department of Chemistry, Michigan State University, East Lansing, Michigan 48824, United States; ‡ Ames National Laboratory, Iowa State University, Ames, Iowa 50011, United States; § Department of Physics and Astronomy, Michigan State University, East Lansing, Michigan 48824, United States; ∥ Department of Physics and Astronomy, Iowa State University, Ames, Iowa 50011, United States; ⊥ Department of Materials Science and Engineering, Iowa State University, Ames, Iowa 50011, United States; # Quantum Measurement Group, MIT, Cambridge, Massachusetts 02139, United States; % Department of Nuclear Science and Engineering, MIT, Cambridge, Massachusetts 02139, United States

## Abstract

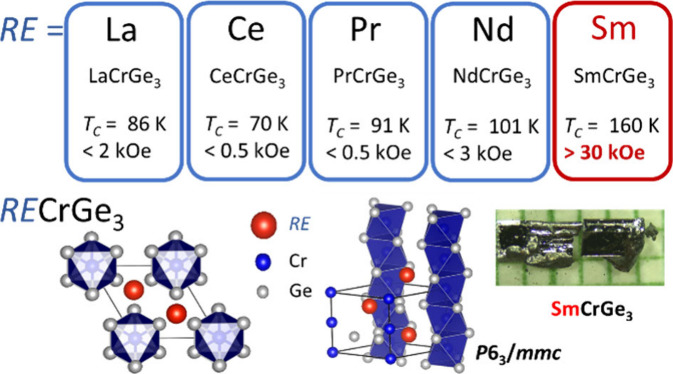

Magnetic anisotropy is a crucial characteristic for enhancing the spintronic device performance. The synthesis of SmCrGe_3_ single crystals through a high-temperature solution method has led to the determination of uniaxial magnetocrystalline anisotropy. Phase verification was achieved by using scanning transmission electron microscopy (STEM), powder, and single-crystal X-ray diffraction techniques. Electrical transport and specific heat measurements indicate a Curie temperature (*T*_C_) of approximately 160 K, while magnetization measurements were utilized to determine the anisotropy fields and constants. Curie–Weiss fitting applied to magnetization data suggests the contribution of both Sm and Cr in the paramagnetic phase. Additionally, density functional theory (DFT) calculations explored the electronic structures and magnetic properties of SmCrGe_3_, revealing a significant easy-axis single-ion Sm magnetocrystalline anisotropy of 16 meV/fu. Based on the magnetization measurements, easy-axis magnetocrystalline anisotropy at 20 K is 13 meV/fu.

## Introduction

Magnetic anisotropy (MA) is a pivotal property of materials that significantly influences the performance of modern spintronic devices.^1^ The interest in MA is attributed to its critical role in various physical phenomena, including the permanent and topological magnets, single-molecule magnets, the Kondo effect, magnetocaloric effects, the magnetic skyrmion dynamics, and so on.^2−7^ Therefore, the large magnetic anisotropy and coercivity are the key properties of functional magnetic materials. From an engineering perspective, though coercivity can be modified by geometrical shaping,^8^ the anisotropy field sets the upper limit for the coercivity. In contrast, from a materials chemistry standpoint, intrinsic magnetocrystalline anisotropy presents a promising avenue for MA control. This intrinsic anisotropy is primarily influenced by the local crystal symmetry and spin–orbit coupling (SOC) associated with the magnetic ions.

Our magnetism research is concentrated on synthesizing novel magnetic materials through the combination of 3*d* transition-metal and 4*f* rare-earth elements, which usually display a range of tunable magnetic functionalities. In many cases of rare-earth and transition-metal (RE–TM) compounds, both 3*d* and 4*f* electrons present magnetic moment and contribute to interesting magnetic properties, such as the high Curie temperature (*T*_C_) and large anisotropy, as in 3*d* compounds, which usually have the high *T*_C_, and the 4*f* compounds, most of which have high magnetocrystalline anisotropy. For example, compounds from the Sm–Co family^9^ and Nd_2_Fe_14_B^9−11^ are prevalent choices within industrial applications due to their substantial contributions to magnetic performance. SmCo_5_ is renowned for its significant magnetocrystalline anisotropy and high coercivity field.^1,10^ In general, then, to achieve industrial applications of magnets, both 3*d* and 4*f* electrons are needed to offer large magnetocrystalline anisotropy and high operation temperature.

*RE*CrGe_3_ compounds, with *RE* representing La, Ce, Pr, Nd, and Sm, crystallize in a hexagonal structure and exhibit intricate magnetic properties due to their unique geometric arrangement of CrGe_6_ clusters and the spatial positioning of Cr and RE atoms.^11−16^ LaCrGe_3_ has garnered extensive investigation owing to its nuanced ferromagnetic properties by varying pressures and distinct magnetic domain behaviors induced by high-field quenching.^12,13,17−22^ Samarium compounds, in particular, display more complex magnetic characteristics due to closely spaced multiplet levels^23^ and the significant orbital contribution of Sm^3+^ ions, which enriches the study of magnetocrystalline anisotropy in permanent magnets anisotropy, necessitating synthesizing its single-crystalline form for detailed analysis.

In this study, we report the synthesis of single-crystalline SmCrGe_3_ and its phase and structural characterization using a comprehensive suite of analytical techniques, including scanning transmission electron microscopy (STEM), energy-dispersive spectroscopy (EDS), powder X-ray diffraction, and single-crystal X-ray diffraction. By conducting magnetization measurements at various temperatures and different magnetic field orientations in conjunction with theoretical calculation, we showed that SmCrGe_3_ possesses a giant easy-axis magnetocrystalline anisotropy, reaching 13 meV/fu at 20 K.

## Experimental Parts and Calculations

### Crystal Growth, Structural Characterization, and Magnetic Measurements

Single crystals of SmCrGe_3_ were synthesized at Ames National Laboratory utilizing a high-temperature solution growth methodology, a technique delineated in refs (13 and 24). This synthesis process entailed a two-step approach. Initially, a mixture consisting of Sm pieces (SM-TWE-0001AM, Ames National Laboratory), Cr pieces (99.996%, Alfa Aesar), and Ge pieces (99.999%, MSE Supplies) with an atomic ratio of 18:12:70 was placed into a Canfield Crucible Set (CCS)^25,26^ and subjected to a thermal regimen where the temperature was increased to 1180 °C. Subsequently, the system underwent a controlled cooling process to 825 °C for 20 h. At 825 °C, a mixture of phases was separated from the liquid by a lab centrifuge. The separated phases are mainly SmCr_0.3_Ge_2_ and CrGe. Second, the decanted liquid was resealed, heated to 850 °C, and slowly cooled from 850 to 810 °C over roughly 15 h. At 810 °C, the growth was decanted, and the SmCrGe_3_ crystalline solid phase with Cr_11_Ge_19_ and Ge single crystals was separated from excess liquid. Since the geometry of different phases is quite different, the SmCrGe_3_ is easily identified and separated from other phases.

Author: To show the crystalline structure and identify potential defects within the SmCrGe_3_ single crystals, a specimen with dimensions of 0.092 × 0.064 × 0.048 mm^3^ was selected for analysis. This crystal was affixed to a nylon loop using Paratone oil, facilitating its examination via a Rigaku XtalLAB Synergy, Dualflex, and Hypix single-crystal X-ray diffractometer. The apparatus was operated at room temperature. Crystallographic data acquisition was conducted employing ω scan methodology, utilizing Mo Kα radiation (λ = 0.71073 Å) emitted from a microfocus sealed X-ray tube under operating conditions of 50 kV and 1 mA. The determination of the experimental parameters, including the total number of runs and images, was derived algorithmically from the strategy computations facilitated by the CrysAlisPro software, version 1.171.42.101a (Rigaku OD, 2023). Subsequent data reduction processes incorporated corrections for Lorentz and polarization effects. Integration of the collected data, predicated on a hexagonal unit cell model, yielded a data set comprising 5510 reflections up to a maximum 2θ angle of 82.192°. Of these reflections, 262 were identified as independent, achieving an average redundancy of 20, with a completeness of 100% and a *R*_int_ value of 7.64%. An advanced numerical absorption correction was implemented, leveraging Gaussian integration across a model of a multifaceted crystal.^27^ Moreover, an empirical absorption correction employing spherical harmonics was applied within the SCALE3 ABSPACK scaling algorithm to refine the data further.^28^Tables S1 and S2 show the results of the single-crystal XRD. The structure was solved and refined using the Bruker SHELXTL software package,^29,30^ using the space group *P*6_3_/*mmc*, with *Z* = 2 for the formula unit, SmCr_0.906(9)_Ge_3_. The final anisotropic full-matrix least-squares refinement on *F*^2^ with 11 variables converged at *R*_1_ = 2.60% for the observed data and *wR*_2_ = 6.72% for all data. The goodness-of-fit was 1.115. The largest peak in the final difference electron density synthesis was 3.87 e^–^/Å^3^, and the largest hole was −2.60 e^–^/Å^3^ with an RMS deviation of 0.402 e^–^/Å^3^. Based on the final model, the calculated density was 7.576 g/cm^3^ and F (000), 359 e^–^.

Powder X-ray diffraction (PXRD) analysis was also performed. The SmCrGe_3_ crystals were grounded using an agate mortar and pestle to achieve a homogeneous powder. This powdered sample was then uniformly distributed on a single crystalline silicon sample holder designed for zero background measurements, with a minimal application of vacuum grease to secure the powder in place. The PXRD data acquisition at room temperature spanned a 2θ range from 15° to 100°, utilizing incremental steps of 0.01° and a fixed dwell time of 3 s per step. These measurements were conducted by using a Rigaku MiniFlex II powder diffractometer, employing Bragg–Brentano geometry coupled with Cu Kα radiation (λ = 1.5406 Å). The refinement of the powder X-ray data was executed using the GSAS-II software suite,^31^ and the occupancy of Cr is 0.931(7).

The phase composition was analyzed by employing a JEOL 6610LV scanning electron microscope equipped with a tungsten hairpin emitter (JEOL Ltd., Tokyo, Japan). For elemental analysis, energy-dispersive X-ray spectroscopy (EDX) was conducted by utilizing an Oxford Instruments AZtec system (Oxford Instruments, High Wycomb, Buckinghamshire, England), operating software version 3.1. This setup included a 20 mm^2^ silicon drift detector (SDD) and an ultrathin window integrated with the JEOL 6610LV SEM. The single crystals of SmCrGe_3_ were affixed to carbon adhesive tape and introduced into the SEM chamber for examination at an accelerating voltage of 20 kV. Data acquisition entailed collecting spectra at multiple points along the individual crystals over an optimized time frame. Quantitative compositional analysis was performed using SEM Quant software, which applies corrections for matrix effects to the intensity measurements. The occupancy of Cr is 0.93(2) as given by the results of EDS, in agreement with the XRD results discussed above. Transmission electron microscope (TEM) samples were prepared with a focused ion beam instrument with a gas injection system (Helios, Thermo Fisher Scientific Ltd.). At room temperature, the TEM samples were investigated by using an aberration-corrected TEM (Titan Cube, Thermo Fisher Scientific Ltd.) at 200 kV.

Temperature- and magnetic-field-dependent DC and VSM magnetization and resistance measurements, as well as temperature-dependent specific heat measurements, were carried out using Quantum Design (QD), Magnetic Property Measurement Systems (MPMS3), and a Physical Property Measurement System (DynaCool). Temperature- and field-dependent DC and VSM magnetization measurements were taken for *H* parallel and perpendicular to the crystallographic *c*-axis by placing the rod-like sample between two collapsed plastic straws with the third, uncollapsed, straw providing support as a sheath on the outside or by using a quartz sample holder. Samples were fixed on a straw or quartz sample holder by GE-7031-varnish. In the VSM magnetization assessments, an oscillation peak amplitude of 4 mm and a mean acquisition time of 2 s were employed to ensure precise data collection. The demagnetizing factor, *N*, is estimated by the dimensions of the sample.^32^*N* < 0.1 as the field is parallel to the *c*-axis, and *N* < 0.5 as the field is perpendicular to the *c*-axis. DC electrical resistance measurements were performed in a standard four-contact geometry using the ACT option of the PPMS. 50 μm diameter Pt wires were bonded to the samples with silver paint (DuPont 4929N) with contact resistance values of about 2–3 ohm. Specific heat capacity measurements under varying temperature conditions were executed by using the relaxation method.

### Theoretical Simulation on Magnetocrystalline Anisotropy

The density functional theory (DFT) calculations are carried out to investigate the band structure and intrinsic magnetic properties of SmCrGe_3_. Although Cr vacancies are present in real samples, our calculations focus on the stoichiometric structure. Experimental lattice parameters and atomic coordinates (Tables S1 and S2) are adopted for the calculations. The calculations are performed using a full-potential linear augmented plane wave (FP-LAPW) method, as implemented in Wien2K.^33^ The generalized gradient approximation of Perdew, Burke, and Ernzerhof^34^ is used for the correlation and exchange potentials. Spin–orbit coupling (SOC) is included using a second variational method. To generate the self-consistent potential and charge, we employed *R*_MT_*·K*_max_ = 8.0 with muffin-tin (MT) radii *R*_MT_ = 2.8, 2.2, and 2.2 au for Sm, Cr, and Ge atoms, respectively. The calculations are performed with 1224 k-points in the irreducible Brillouin zone (IBZ) and iterated until the charge differences between consecutive iterations are smaller than 10^–5^*e* and the total energy differences are lower than 10^–3^ mRy/cell. For the density of states (DOS) calculation, a denser k-mesh with 1710 k-points in the IBZ is used. The strongly correlated Sm-4*f* electrons are treated using the DFT+*U* method with the fully localized-limit (FLL) double-counting scheme. For magnetic properties calculations using DFT-based methods, the orbital dependence of self-interaction error can often contradict Hund’s rules and plague MA calculations. Therefore, the 4*f* configurations of the converged solutions should be carefully monitored to avoid unphysical results. Detailed discussions of challenges and methods of MA calculations can be found in ref (35).

## Results and Discussion

### Hexagonal Perovskite Structure and Defects in SmCrGe_3_

**Figure 1:**
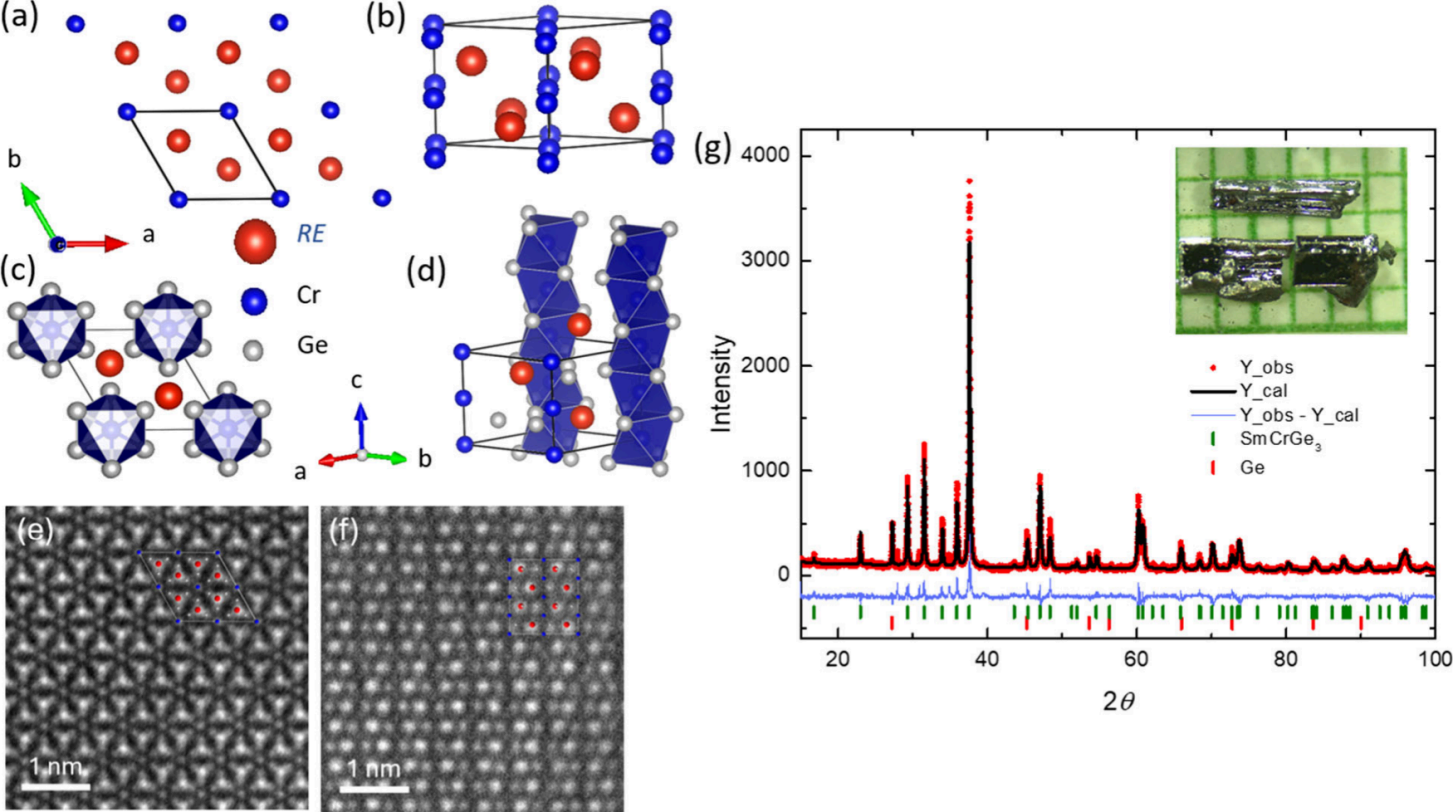
Single-crystal structure and powder X-ray diffraction pattern of SmCrGe_3_. (a, c) Structural viewpoint and (e) high angle annular dark field (HAADF) STEM image from the *c*-axis. (b, d) Structural viewpoint and (f) HAADF STEM image from the *ab*-plane. (g) Power X-ray diffraction data at room temperature with the Rietveld refinement done by GSAS-II shown with the prime phase being SmCr_0.93_Ge_3_ and the minor phase Ge. The red dots indicate the intensity measured, the black line shows the fitting, and the blue line presents the residuals of the fitting. (inset) The crystal picture is over a millimeter paper grid.

Utilizing single crystal X-ray diffraction (SXRD) analysis, we confirm that SmCrGe_3_ crystallizes in a known hexagonal close-packed structure characterized by alternating layers of SmGe_3_ and CrGe_6_ octahedra.^11^ These CrGe_6_ octahedra are uniquely arranged in columns, sharing a pair of opposing faces, forming linear chains of chromium (Cr) atoms along the crystallographic *c*-axis. The interatomic Cr–Cr distance within these chains is measured at 2.83 Å, noticeably shorter than the Cr–Cr separation observed in elemental chromium (2.89 Å). This structural motif is encapsulated within the space group *P*6_3_/*mmc* (No. 194), as shown in the schematic diagram of Figures 1a–1d and the corresponding high-angle annular dark field STEM image of Figures 1e and 1f. ABX_3_ compounds, where A denotes a large cation, B a small cation, and X an anion, are broadly categorized into various structural types based on the stacking sequences of their AX_3_ layers, known as perovskite structures. These include configurations with two-, three-, and six-layer arrangements, among others. In the cubic close-packed variation of these structures, BX_6_ octahedra interconnect exclusively via their vertices. Contrastingly, SmCrGe_3_ exhibits hexagonal close-packing of SmGe_3_ layers, with CrGe_6_ octahedra columnar stacking through face-sharing interactions, as previously mentioned. A comparative analysis reveals that from a purely ionic bonding perspective, SmCrGe_3_’s structural configuration is ostensibly less stable than that of the cubic perovskites due to the significant interionic repulsion prompted by the shortened Cr–Cr distances. It is noted that larger A-site cations (e.g., Ba and La) can mitigate this repulsion by stabilizing the hexagonal close-packing arrangement of the AX_3_ layers, thus favoring the face-sharing geometry of the BX_6_ octahedra. In the context of SmCrGe_3_, the ionic radius of Sm^3+^ (109 pm) is comparatively smaller than that of La^3+^ (117 pm), raising inquiries regarding the mechanism by which Sm^3+^ accommodates such compact Cr–Cr distances. Indeed, SXRD analyses reveal approximately 10% vacancies at the Cr sites, a defect structure that likely ameliorates otherwise pronounced Cr–Cr repulsive interactions. Figure 1g presents the Rietveld refinement of powder X-ray diffraction data, indicating the presence of solely SmCrGe_3_ and minor Ge flux, with an inset displaying a photograph of the single-crystalline specimens, characterized by their metallic luster and bar-like morphology. The details of single-crystal X-ray diffraction measurements are shown in Tables S1 and S2. The occupancy of Cr is 0.906(9). Compared with the occupancy 0.931(7) from power X-ray diffraction and 0.93(2) for EDS (Figure S1), the occupancy number from single-crystal X-ray diffraction is larger than PXRD, which may be due to the measurement conditions and sample variation. However, these data are consistent with EDS results and give more than a 5% vacancy in the Cr site. Instead of using SmCr_0.906_Ge_3_ or SmCr_0.931_Ge_3_, we use SmCrGe_3_ to denote the compound throughout the text.

**Figure 2:**
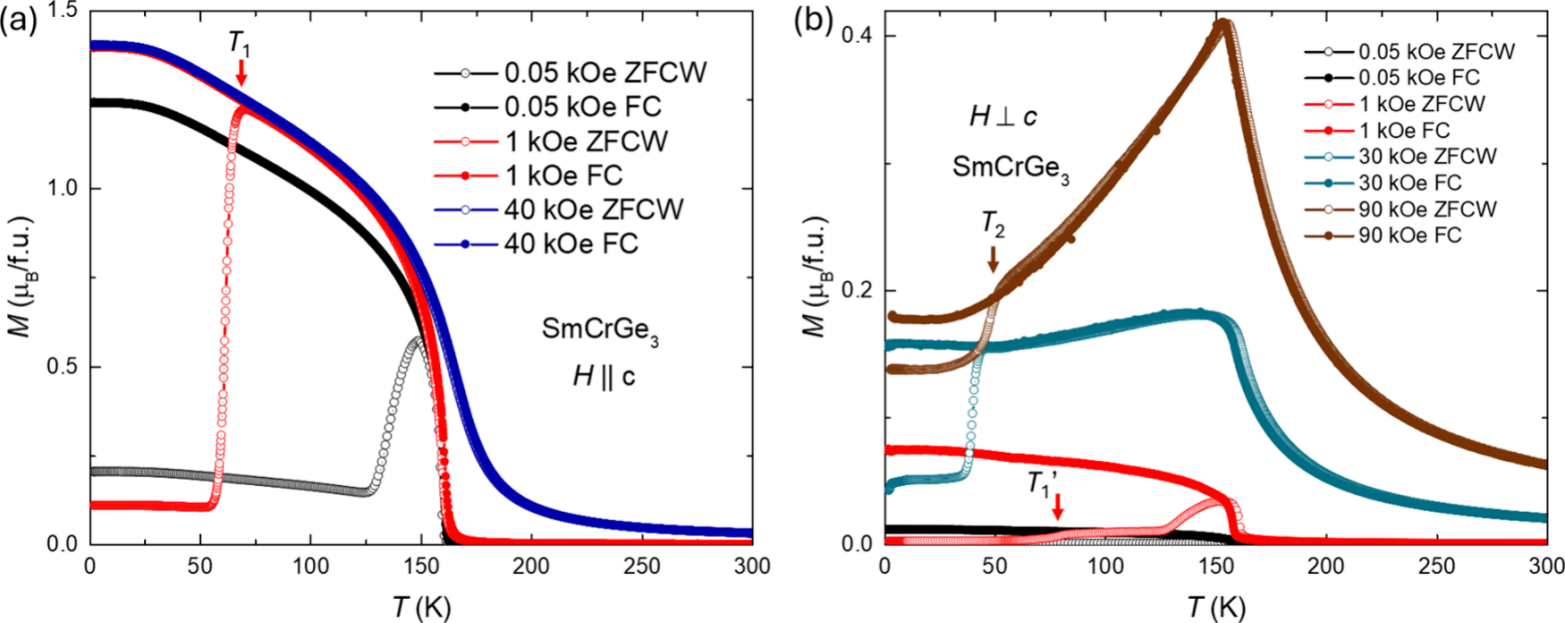
Temperature-dependent magnetization of SmCrGe_3_ measured along different directions at various applied fields in zero-field-cooled-warming (ZFCW) and field-cooled-cooling (FCC) temperature protocols. Figure 2a shows the measurements taken along the *c*-axis. *T*_1_ is the feature temperature that characterizes the jump-like feature as the field is 1 kOe. Figure 2b gives the measurements taken perpendicular to the *c*-axis. *T*_1_′ and *T*_2_ are the feature temperatures that characterize the kink-like and jump features.

Figure 2 presents the analysis of temperature-dependent zero-field-cooled-warming (ZFCW) and field-cooled-cooling (FCC) magnetization measured under various magnetic fields applied parallel or perpendicular to the *c*-axis. Figure S2 presents the *M*(*T*) measurements under more magnetic fields. The ferromagnetic transition is around 159.6 K, which is also evident via the zero-field temperature-dependent electrical transport and specific heat measurements in Figure S4. Figure 2a shows the temperature-dependent magnetization as the field is applied parallel to the *c*-axis, which presents irreversibility at low temperature. The temperature of the jump-like features due to the domain elimination during warming decreases as the magnetic field increases. No hysteresis was observed in Figure 2S with a magnetic field above 30 kOe. As shown in Figure 2b, when the magnetic field is applied in the direction perpendicular to the *c*-axis, several *M*(*T*) differences are observed from the other direction. First, the maximum moment, ∼0.4 μ_B_ (90 kOe), is much smaller than 1.4 μB (30 kOe) in *H*||*c*. Second, the jump features are also observed, but these features still exist in high magnetic fields, which may be due to a few degrees of sample misalignment relative to the magnetic field direction. The kink-like features, shown as *T*_1_′, appear at similar temperatures as *T*_1_. Finally, the decrease in the moment as the temperature decreases appears after the transition with the high magnetic field in *H*⊥*c*. There is no confirmed explanation for the moment decrease under a magnetic field larger than 16 kOe, as shown in Figures 2Sb and 2Sd. There is one suspicion for magnetic moment decrease. This decrease in the nondomain-motion range (the range ZFCW and FCC overlap) indicates no domain change. The magnetization decrease means that the spins move away from the magnetic field direction and rotate to the easy direction. This is the competition between the Zeeman interaction and the magnetocrystalline anisotropy. The different magnetization responses to magnetic fields applied parallel versus perpendicular to the *c*-axis further accentuate the anisotropic nature of the material.

**Figure 3:**
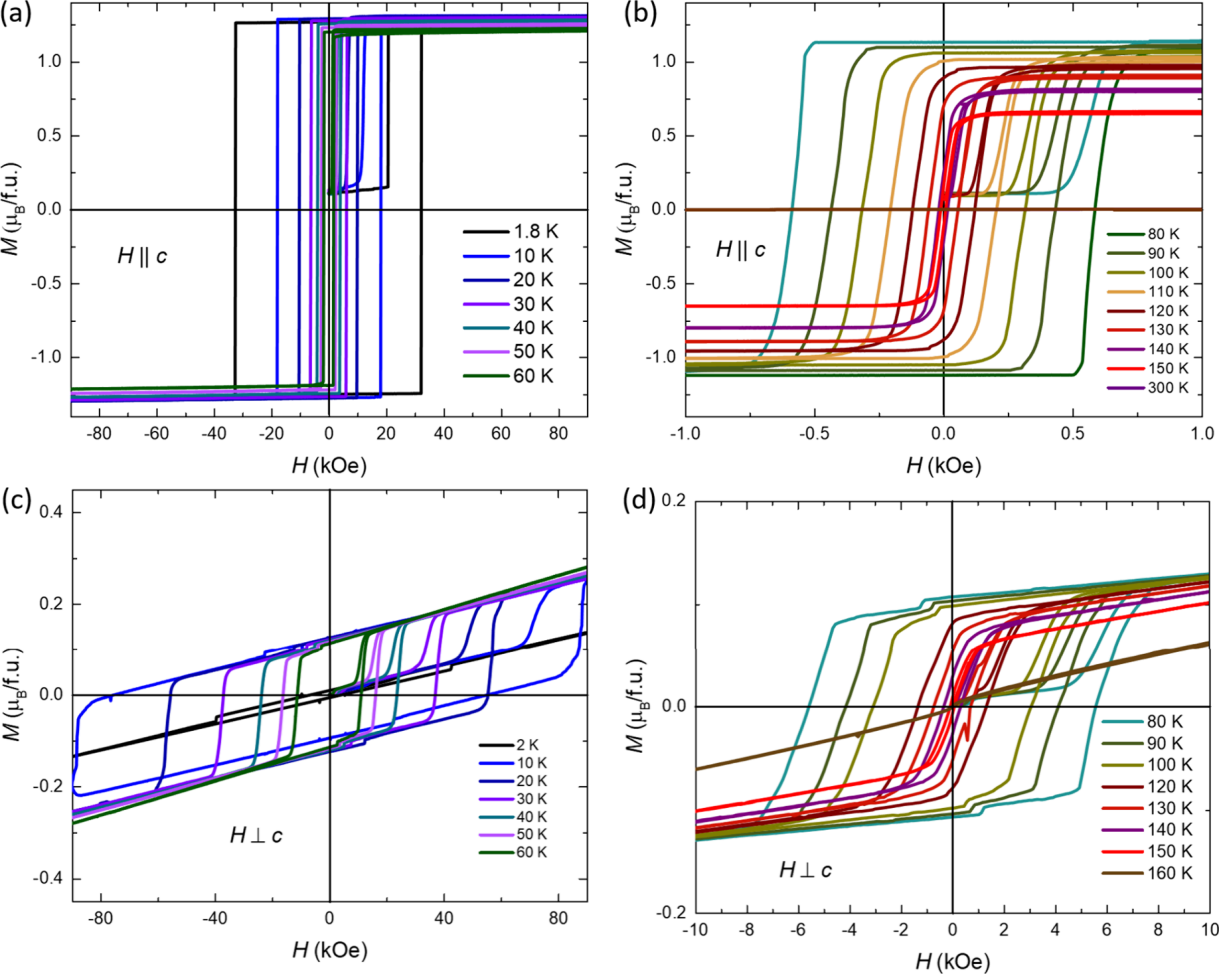
Field-dependent magnetization measured at various temperatures along different directions. Magnetization of a single crystal of SmCrGe_3_ at different temperatures as a function of the magnetic field applied parallel (a, b) or perpendicular (c, d) to the crystallographic *c*-axis. Each isothermal loop is a 5-quadrant loop. Between loops, the system is taken to 300 K and then cooled to zero field to the next temperature. Since the no-zero remnant field exists, the moment at zero field in the first quadrant is not zero.

Figure 3 presents the field-dependent magnetization of an SmCrGe_3_ single crystal at various temperatures. The low-temperature magnetization loop, when the magnetic field is aligned parallel to the *c*-axis, indicates a hard ferromagnetic material. At a given temperature and with increasing field, the distribution of domains with different orientations does not change until a certain saturation field is achieved. As shown in Figure S3a, even with a log scale, the width of the transition to saturation is very small in the low-temperature range. At 1.8 K, the loop exhibits the highest coercivity of approximately 30 kOe. The coercivity decreases with increasing temperature, accompanied by a gradual departure from the loop’s initial rectangular configuration at high temperatures. The saturation magnetic moment is around 1.26 μ_B_, which is smaller than 1.4 μ_B_ in the temperature-dependent magnetization measurements at 40 kOe. The discrepancy in saturation moments may be due to differences in the experimental setups. In field-dependent magnetization measurements, the sample was mounted on a straw instead of a quartz rod, which prevented it from falling over a long period in high magnetic field. When the magnetic field is applied perpendicular to the *c*-axis, the magnetic moment does not reach saturation even at fields up to 90 kOe. Except for the jump features of magnetization above 1.8 K, the kink features are also observed, forming a small hysteresis at 1.8 K. The reason for the kink features appearing in Figure 3d is not known. The larger hysteresis shown above 1.8 K could be due to the small misalignment perpendicular to the *c*-axis, which should be the projection of easy-axis moments.

**Figure 4:**
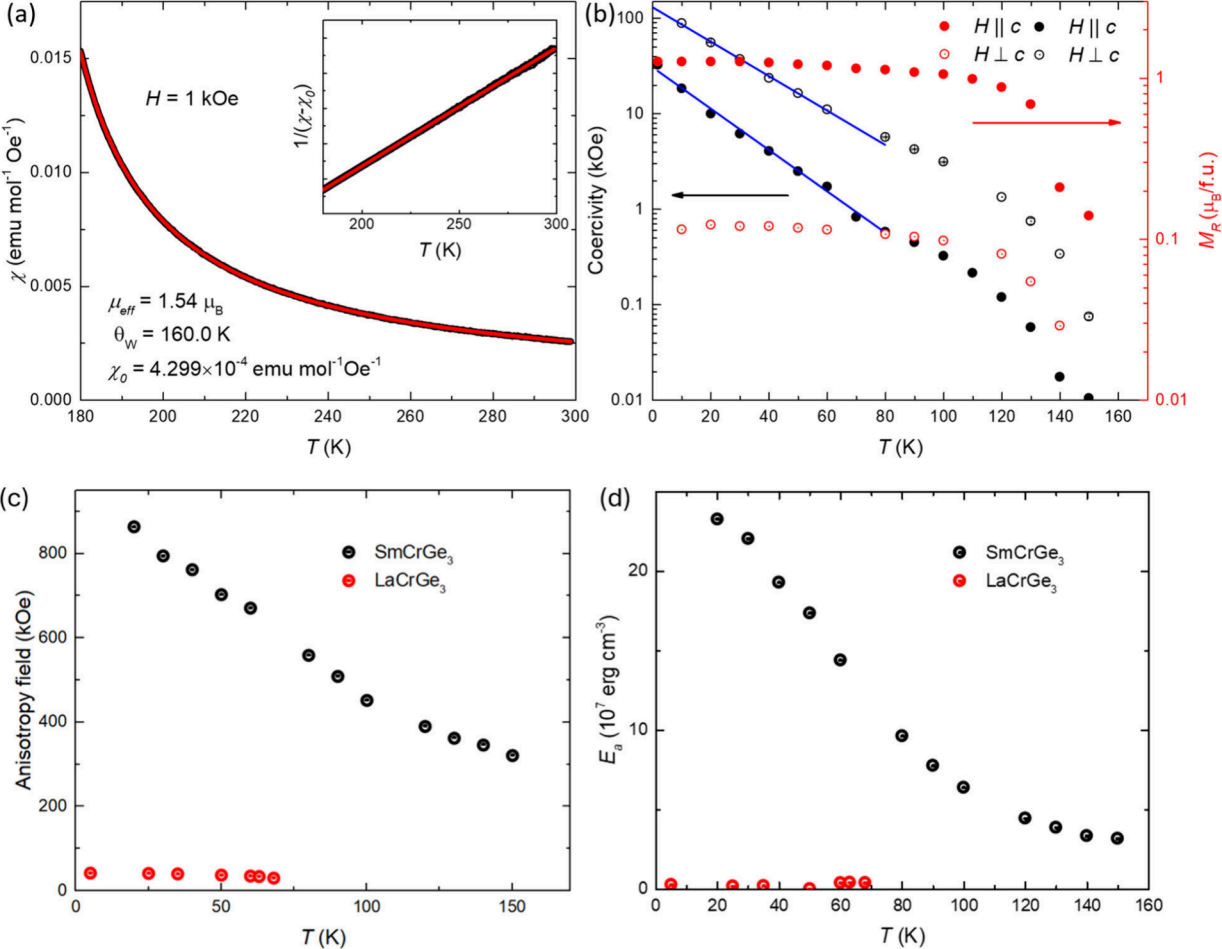
Curie–Weiss analysis, coercivity field (black), remanent magnetization (red), anisotropy energy, and *M*(*H*) in different θ (the angle between the *c*-axis and the magnetic field). Panel (a) shows the Curie–Weiss fitting (χ = *C*/(*T* – θ_W_) + χ_0_) applied on the temperature range from 180 to 300 K. Inset presents a linear fit to 1/(χ – χ_0_) in a function of temperature. The coercivity field (black) and remanent magnetization (red), *M*_*R*_, as a function of temperature, are shown in (b) as the magnetic field parallel (solid) and perpendicular (hollow) to the crystallographic *c*-axis in the log scale. The blue line shows the linear fit on the log scale data. Panel (c) shows the anisotropy fields of SmCrGe_3_ (black) and LaCrGe_3_ (red) as a function of temperature. Panel (d) presents the anisotropy energy, *E*_a_, of SmCrGe_3_ (black) and LaCrGe_3_ (red) as functions of temperature.

The Curie–Weiss model, characterized by the equation χ = *C*/(*T* – θ_W_) + χ_0_, has been employed to fit the magnetization–temperature (*M*(*T*)) data under an applied magnetic field of 1 kOe for polycrystalline average data for SmCrGe_3_, as depicted in Figure 4a. Here, χ represents the magnetic susceptibility, *C* is the Curie constant, *T* is the temperature, θ_W_ is the Weiss temperature, and χ_0_ is a temperature-independent susceptibility term. The analysis incorporates a polycrystalline average susceptibility calculated as χ = (2χ_⊥_ + χ_∥_)/3, where χ_⊥_ and χ_∥_ denote susceptibilities perpendicular and parallel to the *c*-axis, respectively. The fitting yields an effective magnetic moment (μ_eff_) of approximately 1.54 μ_B_ and a Curie–Weiss temperature (θ_W_) of 160.0 K. The θ_W_ value is very close to the ferromagnetic transition temperature identified through heat capacity measurements, as shown in Figure S4. Compared with the theoretical Sm^3+^ value of 0.84 μ_B_,^36^ the fitting effective moment of SmCrGe_3_ is 1.54 μ_B_/formula. Considering results from other RECrGe_3_ compounds,^14−16,37^ it is difficult to determine if both 3*d* and 4*f* electrons contribute to the effective moments. Compared to LaCrGe_3_, which has an effective moment of 2.5 μ_B_/fu^12^ due to spin fluctuations near the transition in the itinerant system,^38,39^ SmCrGe_3_ likely exhibits more complex contributions to the magnetization tail. These contributions may arise from both the localized moments of 4*f* and itinerant electrons. The decrease of the SmCrGe_3_ effective moment after replacing La with Sm may be due to the potential change of the electronic band structure and the mixed valence of Sm.

The coercivity, a key magnetic property indicative of the resistance to demagnetization, of SmCrGe_3_ exhibits a pronounced temperature dependence in both orientations relative to the crystallographic axes, as presented in Figure 4b. Notably, as the linear fitting shown in a semilog plot, the coercivity decreases exponentially as the temperature increases to 80 K, suggesting a change from high to low pinning strength with increasing temperature. Figure S3 elucidates these distinctions by comparing the virgin curves of SmCrGe_3_ and LaCrGe_3_ single crystals with the applied magnetic field parallel to the *c*-axis. Both materials exhibit rectangular hysteresis loops at lower temperatures, indicating their ferromagnetic nature. Nevertheless, the critical field required to saturate the magnetization of SmCrGe_3_ surpasses that of LaCrGe_3_ by several orders of magnitude. Specifically, according to Figure S3, a 0.2 kOe is sufficient to align the magnetic domains in LaCrGe_3_ as the field along the *c*-axis, whereas SmCrGe_3_ necessitates a magnetic field magnitude hundreds of times greater to achieve domain alignment. This comparison distinguishes SmCrGe_3_ as a hard ferromagnet due to magnetocrystalline anisotropy induced by Sm, in contrast to the intrinsically soft ferromagnetic nature of LaCrGe_3_, highlighting the substantial variation in its magnetic domain behavior and coercivity.

To further study the magnetic anisotropy, the anisotropy field of SmCrGe_3_ with a comparison to LaCrGe_3_ as a function of temperature is presented in Figure 4c, estimated by extrapolating the magnetization–field (*M*(*H*)) curves in the high-field regime after jump-like features, with the magnetic field applied perpendicular to the *c*-axis. Details of plotting Figures 4c and 4d are shown in Figure S6. The evaluation of the magnetic anisotropy within a hexagonal crystal system is quantified by the anisotropy constants, considering the first two anisotropy constants for the simplicity, *K*_1_ and *K*_2_, according to the expression for anisotropy energy (*E*_a_):^40^
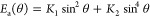
1The relation for the anisotropy field (*H*) per magnetization component perpendicular to the *c*-axis (*M*_*ab*_) is given by^40^
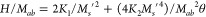
2Here, *E*_a_(θ) represents the anisotropy energy, θ is the angle between the magnetization vector and the *c*-axis, *M*_*ab*_ signifies the magnetization component perpendicular to the *c*-axis, and *M*′ denotes the spontaneous magnetization.^40^ The analysis employs saturated magnetization for fitting purposes, ensuring that the fitting is conducted in the high-field domain, where magnetic domains are predominantly aligned in a singular direction. As demonstrated in Figure 4d, the anisotropy energy *E*_a_ for SmCrGe_3_ significantly surpasses that of LaCrGe_3_, indicating a remarkable enhancement in magnetic anisotropy attributed solely to the presence of Sm. This significant disparity underscores the critical role of Sm in enhancing the magnetic anisotropy in SmCrGe_3_, delineating a stark contrast in the magnetic properties of these two compounds. In linear theory^41^
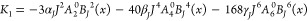
3
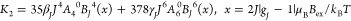
4*B*_ex_ is the exchange field acting on the rare-earth sublattice, *B*_*J*_^*n*^(*x*) is the generalized Brillouin function, *A*_*n*_^0^ is the uniaxial crystal field parameters, and α_*J*_, β_*J*_, and γ_*J*_ are the Stevens coefficients.^22,41^ According to *K*_1_ and *K*_2_ expressions, even considering the higher-order and *J* mixing,^41^ the 3*d*–4*f* exchange interaction plays an important role in magnetocrystalline anisotropy. This explains the significant increase in anisotropy of SmCrGe_3_ compared with that of LaCrGe_3_.

Table 1 summarizes the magnetic moments and their components in SmCrGe_3_ calculated in DFT+*U* with various *U* values. Here, the signs of the magnetic moments indicate their directions. Note that according to Hund’s rules, Sm^3+^ has a configuration with *S* = 5/2, *L* = 5, and *J* = 5/2. For smaller *U* values, the calculated 4*f* spin and orbital magnetic moments, *m*_4*f*_^*l*^ and *m*_4*f*_^x*s*^, respectively, show a large deviation from the integer numbers. This is caused by the pining of Sm states at the Fermi level unless a sufficiently large *U* is applied on Sm-4*f* orbitals in DFT+*U*. As a result, the shoulder of the Sm states right above the Fermi level is slightly filled up, resulting in more than five electrons, e.g., ∼5.18 *e* at *U* = 6 eV, occupying the Sm-4*f* states. A *U* value larger than 10 eV is required to push the unoccupied 4*f* states away from *E*_F_, ensuring a Sm^3+^(*f*^5^) configuration. Therefore, in the following we mainly discuss the calculations performed with *U* = 12 eV.

**Table 1:** Total Magnetic Moment *M*, On-Site Sm and Cr Magnetic Moments, *m*_Sm_ and *m*_Cr_, in SmCrGe_3_ Calculated with Various *U* Values Applied on Sm-4*f* Orbitals in DFT+*U*^a^

*U*	*m*_4*f*_^*l*^	*m*_4*f*_^*s*^	*m*_Sm_^*s*^	*m*_Sm_	*m*_Cr_	*M*
6	4.17	–5.18	–5.34	–1.17	1.58	0.35
8	4.53	–5.11	–5.25	–0.72	1.54	0.74
10	4.81	–5.00	–5.18	–0.37	1.51	1.05
12	4.92	–4.97	–5.16	–0.24	1.51	1.18
14	4.95	–4.97	–5.16	–0.21	1.52	1.21

a The Sm magnetic moment is further resolved into its spin contribution *m*_Sm_^*s*^ and the contributions from Sm-4*f* spin and orbital, *m*_4*f*_^*s*^ and *m*_4*f*_^*l*^. The total magnetization *M* is in units of μ_B_/fu, while all other components are in units of μ_B_/atom and calculated inside the muffin-tin (MT) spheres. The magnetic moment of Ge is negligible (∼0.02 μ_B_/Ge) and not listed. The Cr orbital magnetic moment is negligible compared with its spin moment. The Sm spin moment and Cr magnetic moment have opposite signs, indicating an antiferromagnetic coupling between the Sm and Cr spins. The opposite signs of the Sm-4*f* orbital and spin magnetic moments reflect the third Hund’s rule for the light rare-earth elements. In the large-*U* limit, the Sm-4*f* total magnetic moment vanishes as its spin and orbital components cancel out, resulting in a small total magnetic moment for Sm (*m*_Sm_), which is primarily due to the 5*d* spin moments that are parallel to the 4*f* spin moments.

**Figure 5:**
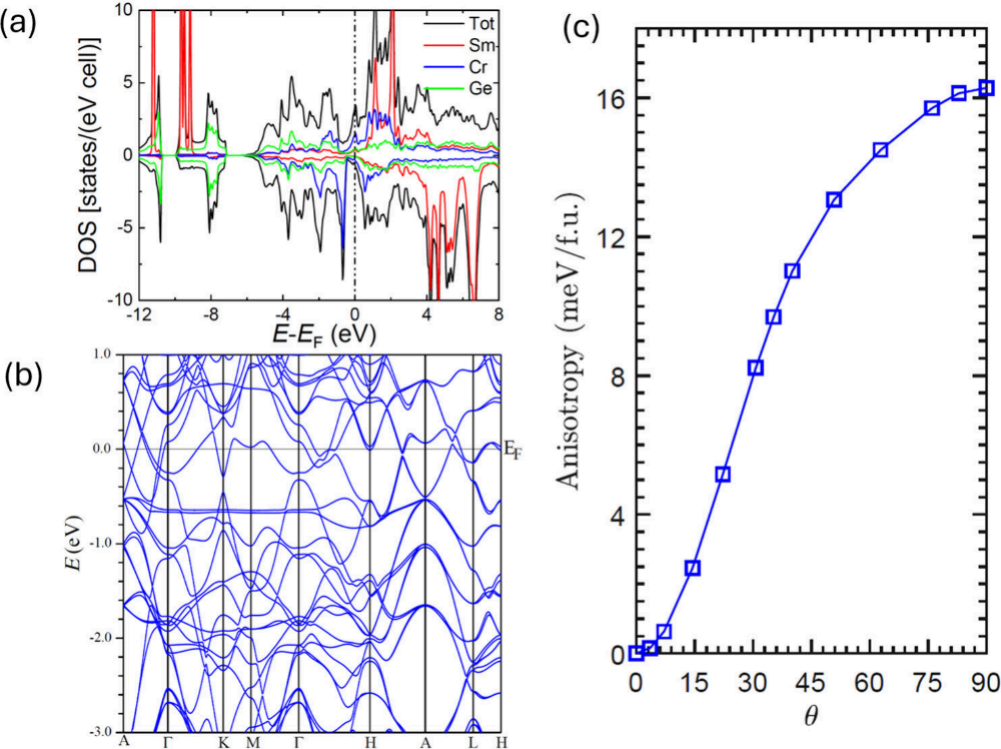
DOS, band structure, and magnetocrystalline anisotropy of SmCrGe_3_, calculated in DFT+*U* with *U* = 12 eV applied to Sm 4*f* states. (a) Total and sublattice-decomposed DOS. The total DOS consists of contributions from all atomic sites and the interstitial region. The unit cell contains two formula units. (b) Band structure along high symmetry directions. Spin–orbit coupling is included in the calculation. A pair of closely aligned flat bands along the Γ–*Κ*–*Μ*–Γ is situated at around −0.7 eV below the Fermi level. These flat bands become dispersive at finite *k*_*z*_. (c) Magnetocrystalline anisotropy, characterized by the variation of magnetic energy (in meV/fu) as a function of spin-axis rotation. The spin direction is denoted by polar angle θ and azimuthal angle ϕ. The lattice vector *c* ([001]) direction is along the *ẑ* direction and is denoted by θ = 0°, while the lattice vector *a* ([100]) direction is denoted by θ = 90° and ϕ = 0°. The calculations are performed with ϕ fixed at 60°. A large easy-axis magnetocrystalline anisotropy energy of ∼16 meV/fu is found.

Figure 5a shows the total and sublattice-decomposed DOS. At *U* = 12 eV, the occupied Sm-4*f* states are located around −10 eV below *E*_F_, while the unoccupied majority-spin 4*f* states are in the range 1–2 eV above *E*_F_, with a negligible shoulder at *E*_F_, resulting in a 4*f*^5^ configuration. The occupied states between −6 eV and *E*_F_ are mainly Ge-4*p*, Cr-3*d*, and Sm-5*d* states, with expected hybridization between them. The Ge-4*s* states are present around the −12 to −8 eV region. The Cr-3*d* minority-spin channel shows a peak at *E*_F_, and its majority-spin channel has a larger sharp peak at ∼0.7 eV below *E*_F_, attributed to a flat band as discussed below. Figure 5b shows the electronic band structure of SmCrGe_3_ calculated along high symmetry directions, primarily in the *k*_*z*_ = 0 and 0.5 (2π/*c*) planes. Noticeably, two seemingly parallel flat bands are around −0.7 eV in the Γ–*Κ*–*Μ*–Γ direction. These two bands are dominated by Cr-*d*_*xy*_ and Cr -*d*_*xz*_ characteristics. The seemingly universal small splitting between them along Γ–*Κ*–*Μ*–Γ is actually caused by the combination of the crystal field and SOC. In the absence of SOC, these two bands degenerate at high symmetric *k* points and slightly split elsewhere. SOC further lifts the band degeneracy, especially by introducing the largest splittings at these otherwise-degenerate high symmetric *k*-points, resulting in overall two seemingly parallel flat bands. It is worth noting that a previous study has reported flat bands at −0.15 eV below *E*_F_ and has attributed it to be the source of the magnetic fragility of LaCrGe_3_.^22^ However, the flat bands in SmCrGe_3_ that we found here are much farther away from *E*_F_, unlikely to play a similar role as reported in LaCrGe_3_. On the other hand, unlike La, Sm-4*f* spin can facilitate Cr spin polarization via the exchange coupling mediated by Sm-5*d* electrons.^42^Figure 5c shows the calculated total energies *E*(θ) as functions of the spin-quantization direction, characterized by the polar angle θ and the azimuthal angle ϕ. The spin axis rotates from the [001] direction to the [110] direction, showing uniaxial anisotropy. The energy minimum occurs at [001], suggesting an easy-axis anisotropy in SmCrGe_3_. The calculated uniaxial anisotropy of *K*_*U*_ = 16 meV/fu is in reasonable agreement with the experimental value of 13 meV/fu (measured at 20 K and *K*_*U*_ = *K*_1_ + *K*_2_) estimated from magnetization measurements. Moreover, the out-of-plane energy profile curve deviates from a sinusoidal shape, suggesting the significance of higher-order crystal parameters in contributing to the magnetic anisotropy.^42^

## Conclusions

Hexagonal SmCrGe_3_ is characterized by significant magnetocrystalline anisotropy, with density functional theory (DFT) calculations revealing an enhancement of magnetocrystalline anisotropy of about 16 meV/fu at 0 K. This value closely aligns with experimental observations, which document an anisotropy of 13 meV/formula unit (fu) at 20 K, comparable to that of SmCo_5_ (13–15 meV/fu at base temperature^43^). The synthesis of SmCrGe_3_ single crystals is achieved through the flux growth method, with subsequent magnetic and specific heat measurements delineating a ferromagnetic transition temperature of approximately 160 K compared with a *T*_C_ of 155 K for the polycrystalline sample.^11^ These studies highlight the influence of 4*f* and 3*d* electrons on the critical contribution toward the noted magnetic anisotropy due to the exchange interaction and crystal field.

## Data Availability

Deposition Number 2374310 contains the supplementary crystallographic data for this paper. These data can be obtained free of charge via the joint Cambridge Crystallographic Data Centre (CCDC) and Fachinformationzentrum Karlsruhe Access Structures service.

## Supporting Information Available

Crystal structure refinement data, atomic coordinates, isotropic displacement parameters, SEM and EDS results, transition temperature values and magnetic fields, transition temperatures in resistance and specific heat, and phase diagram of transition temperatures (PDF)
