# Supplementary material for: Giant Uniaxial Magnetocrystalline Anisotropy in SmCrGe3
Source: J Am Chem Soc. 2024 Oct 28;146(44):30294–302. doi: 10.1021/jacs.4c10056 (PMC11544618; doi:10.1021/jacs.4c10056)
Supplement: Supplementary file 1 — ja4c10056_si_001.pdf [file ja4c10056_si_001.pdf]

Support Information

Giant Uniaxial Magnetocrystalline Anisotropy in $SmCrGe_3$

Mingyu Xu,[1] Yongbin Lee[2], Xianglin Ke,[3] Min-Chul Kang,[2] Matt Boswell,[1] Sergey. L. Bud'ko,[2, 4] Lin Zhou,[2, 5] Liqin Ke[2], Mingda Li,[6,7] Paul. C. Canfield,[2, 4*] Weiwei Xie[1*]

[1]Department of Chemistry, Michigan State University, East Lansing, MI 48824, USA
[2]Ames National Laboratory, Iowa State University, Ames, IA 50011, USA
[3]Department of Physics and Astronomy, Michigan State University, East Lansing, MI 48824, USA
[4]Department of Physics and Astronomy, Iowa State University, Ames, IA 50011, USA
[5]Department of Materials Science and Engineering, Iowa State University, Ames, IA 50011, USA
[6]Quantum Measurement Group, MIT, Cambridge, MA 02139, USA
[7]Department of Nuclear Science and Engineering, MIT, Cambridge, MA 02139, USA

Table S1. Crystal structure refinement data……………………………………………………...S2

Table S2. Atomic coordinates and isotropic displacement parameters……................................S2

Fig. S1. SEM and EDS results…………………….……………………………………..S3

Fig. S2. Transition temperature values and magnetic fields…...……………………………….S4

Fig. S3. First-quadrant field-dependent magnetization. ………………………………………S6

Fig. S4. Temperature-dependent specific heat...………………………………………………S7

Fig. S5. Phase diagram of T_1, T_1' and T_2…...……………………………………………………S8

Fig. S6. Anisotropy field and anisotropy energy fitting …………………………………... S10

Table S1. The crystal structure and refinement of $SmCrGe_3$ at room temperature K (Mo Kα radiation). Values in parentheses are estimated standard deviation from refinement.

**Chemical Formula**	$\mathbf{SmCr_{0.89}Ge_3}$
Formula Weight	415.18 g/mol
Space Group	$P6_3/mmc$
Unit Cell dimensions	a = 6.0898(3) Å<br>b = 6.0898(3) Å<br>c = 5.6666(3) Å
Volume	181.99(2) $Å^3$
Z	2
Density (calculated)	7.576 g/cm^3
Absorption coefficient	43.109 mm^{-1}
F (000)	359
2θ range	7.728 to 82.192°
Reflections collected	5510
Independent reflections	262 [R_{int} = 0.0764]
Refinement method	Full-matrix least-squares on F^2
Data/restraints/parameters	262/0/11
Final R indices	R_1 (I>2σ(I)) = 0.0253; wR_2 (I > 2 σ(I)) = 0.0662<br>R_1 (all) = 0.0260; wR_2 (all) = 0.0672
Largest diff. peak and hole	+3.87 $e^-/Å^3$ and -2.60 $e^-/Å^3$
R. M. S. deviation from mean	0.402 $e^-/Å^3$
Goodness-of-fit on F^2	1.115

Table S2. Atomic coordinates and equivalent isotropic atomic displacement parameters ($Å^2$) of $SmCrGe_3$. (U_{eq} is defined as one-third of the trace of the orthogonalized U_{ij} tensor.)

$\mathbf{SmCr_{0.89}Ge_3}$	**Wyck.**	***x***	***y***	***z***	**Occ.**	$\mathbf{U_{eq}}$
**Sm**	2*d*	1/3	2/3	3/4	1	0.006(1)
**Ge**	6*h*	0.1931(1)	0.3862(1)	1/4	1	0.005(1)
**Cr**	2*a*	0	0	0	0.906(9)	0.005(1)

Tables S1 and S2 show the results of the single-crystal XRD. The structure was solved and refined using the Bruker SHELXTL Software Package with the space group *P6₃/mmc*, $SmCr_{0.906(7)}Ge_3$. The final anisotropic full-matrix least-squares refinement on F^2 with 11 variables converged at R_1 = 2.60 %, for the observed data and wR_2 = 6.72 % for all data. The goodness-of-fit was 1.115. The largest peak in the final difference electron density synthesis was 3.87 $e^-/Å^3$, and the largest hole was -2.60 $e^-/Å^3$ with an RMS deviation of 0.402 $e^-/Å^3$. Based on the final model, the calculated density was 7.576 g/cm^3 and F (000), 359 e^-.

a)

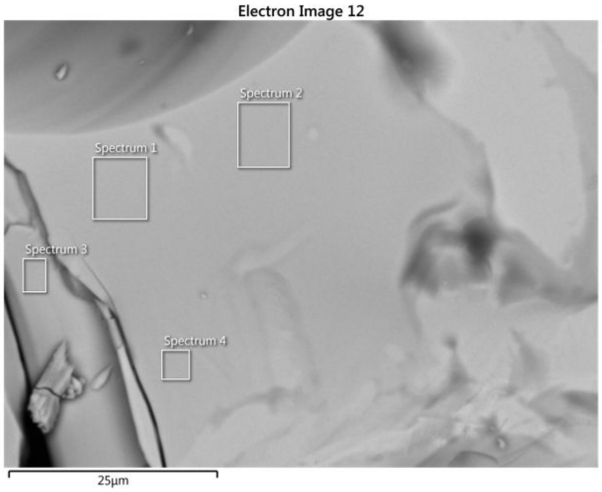

b)

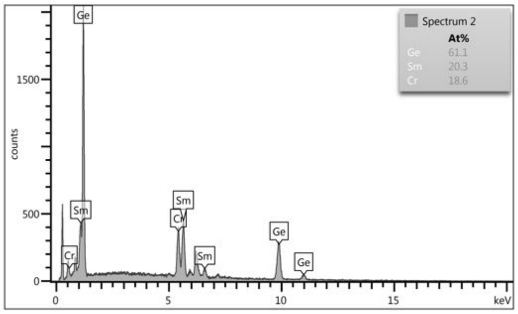

c)

Sm	1
Cr	0.93(2)
Ge	2.97(7)

Fig. S1. SEM and EDS results of single crystals SmCrGe3. **Fig. S1*a*** gives the SEM image of one of the measured samples. **Fig. S1*b*** shows one of the EDS spectrum results. **Fig. S1*c*** presents the statistical results of 13 spectra from two different pieces of samples.

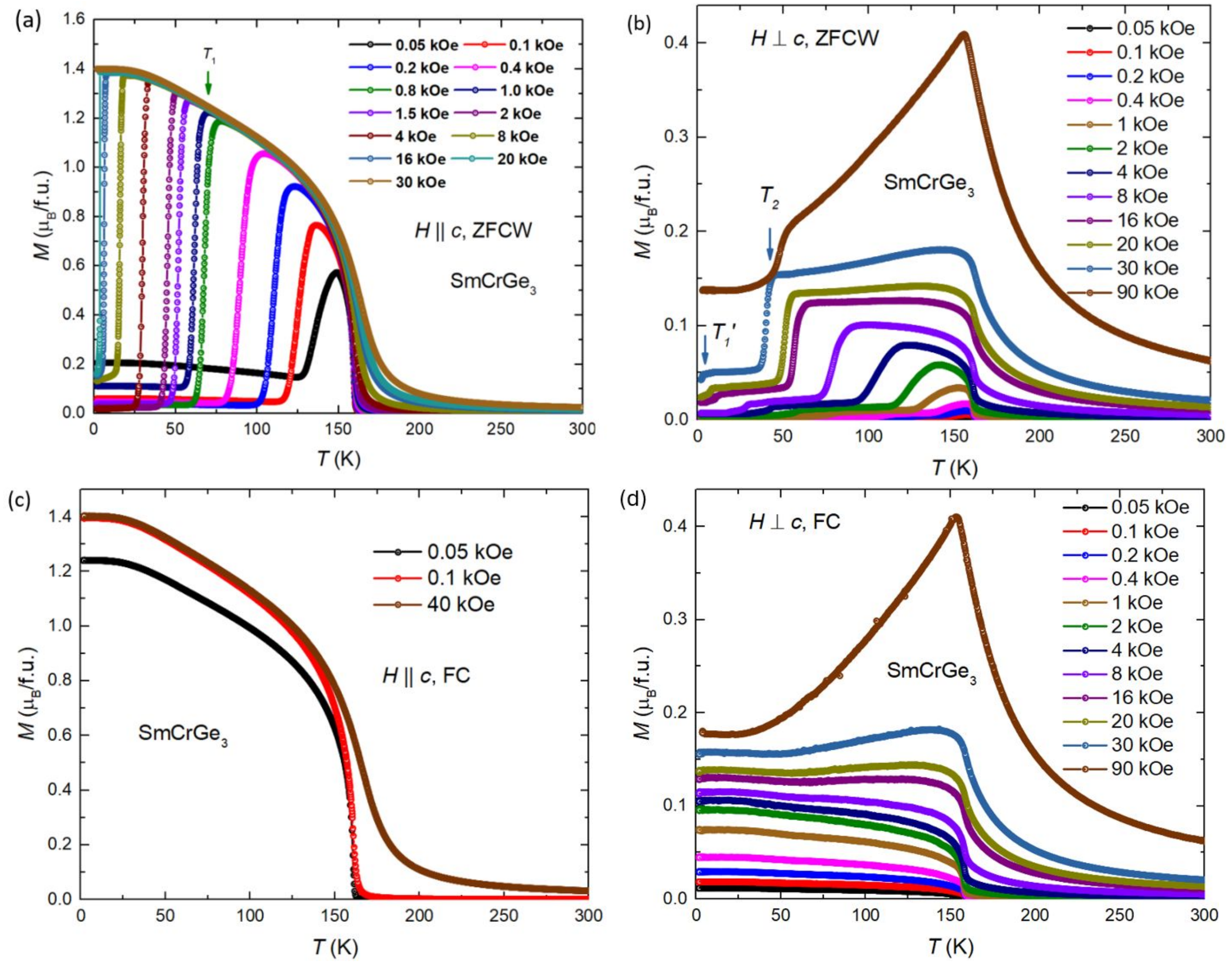

Fig. S2. Temperature-dependent magnetization of $SmCrGe_3$ measured along different directions at various applied fields in zero-field-cooled-warming (ZFCW) and field-cooled-warming mode. (**Fig. S2*a***) measurements conducted along the *c*-axis in ZFCW. T_1 is the feature temperature that characterizes the jump-like feature as the field is 0.8 kOe. (**Fig. S2*b***) measurements are conducted perpendicular to the *c*-axis in ZFCW. T_1' and T_2 are the feature temperatures that characterize the kink-like features as the field is 30 kOe. (**Fig. S2*c***) measurements conducted along the *c*-axis in FC. (**Fig. S2*d***) measurements conducted perpendicular to the *c*-axis in FC.

Fig. 2S*a* and **2S*c*** show the $M(T)$ under different magnetic fields parallel to the *c*-axis under the ZFCW and FC protocol. As the field increases, the jump-like features in ZFCW measurements, denoted as T_1, are suppressed as the magnetic field increases. When the magnetic field increases to 30 kOe, the jump-like features disappear, and there is no hysteresis. **Fig. 2S*b*** shows the temperature-dependent magnetization with a field perpendicular to the *c*-axis under the ZFCW temperature protocol. As shown in the 30 kOe magnetization data, two kink-like features are shown and denoted as T_1' and T_2. Both of these feature temperatures

decrease as the magnetic field increases. As the magnetic field is smaller than 8 kOe, the magnetization increases after transition, then decreases around T_2. When the magnetic field is larger than 16 kOe, the magnetization directly decreases after the transition. This drop of magnetization before T_2 becomes larger as the magnetic field increases. When the magnetic field reaches 90 kOe, this decreased value becomes significant and reaches almost half the maximum magnetization near the transition temperature. **Fig. 2S*c*** gives magnetization as a function of temperature in the magnetic field parallel to the *c*-axis under FC. Below 0.1 kOe, the magnetization at the base temperature increases as the magnetic field increases. After 0.1 kOe, there is no observable magnetization change at base temperature as the field increases. **Fig. 2S*d*** shows the temperature-dependent magnetization in the magnetic field up to 90 kOe perpendicular to the *c*-axis under FC. When the magnetic field is below 8 kOe, the magnetization increases as the temperature decreases and reaches maximum at the base temperature. When the applied field is larger than 16 kOe, after transition, the magnetization decreases first, then increases a little. As the magnetic field goes up to 90 kOe, the magnetization significantly decreases as the temperature decreases. In **Fig. 2S*a***, the magnetization value reaches around 1.4 μ_B at 30 kOe when the field is parallel to the *c*-axis; however, when the field is perpendicular to the *c*-axis, even the magnetic field is three times larger than 30 kOe, the maximum magnetization is only less than half of the magnetization in another direction. This indicates the magnetocrystalline anisotropy in $SmCrGe_3$.

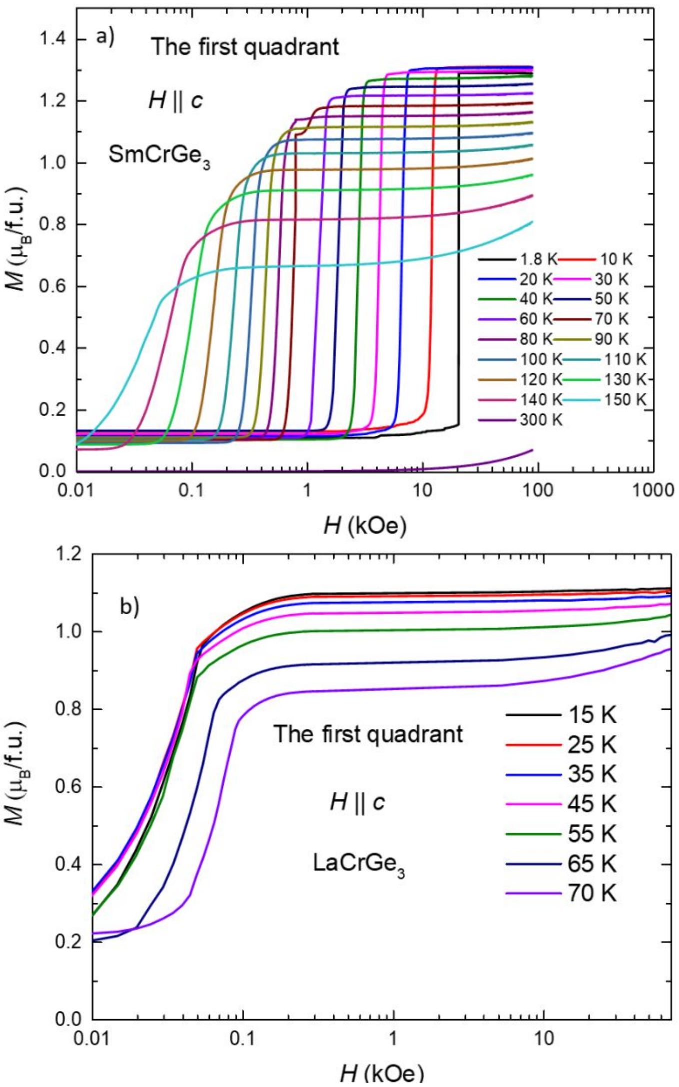

Figure S3. First-quadrant field-dependent magnetization. The first-quadrant field-dependent magnetization is plotted for both $SmCrGe_3$ and $LaCrGe_3$ single crystal as a field parallel to the *c*-axis.

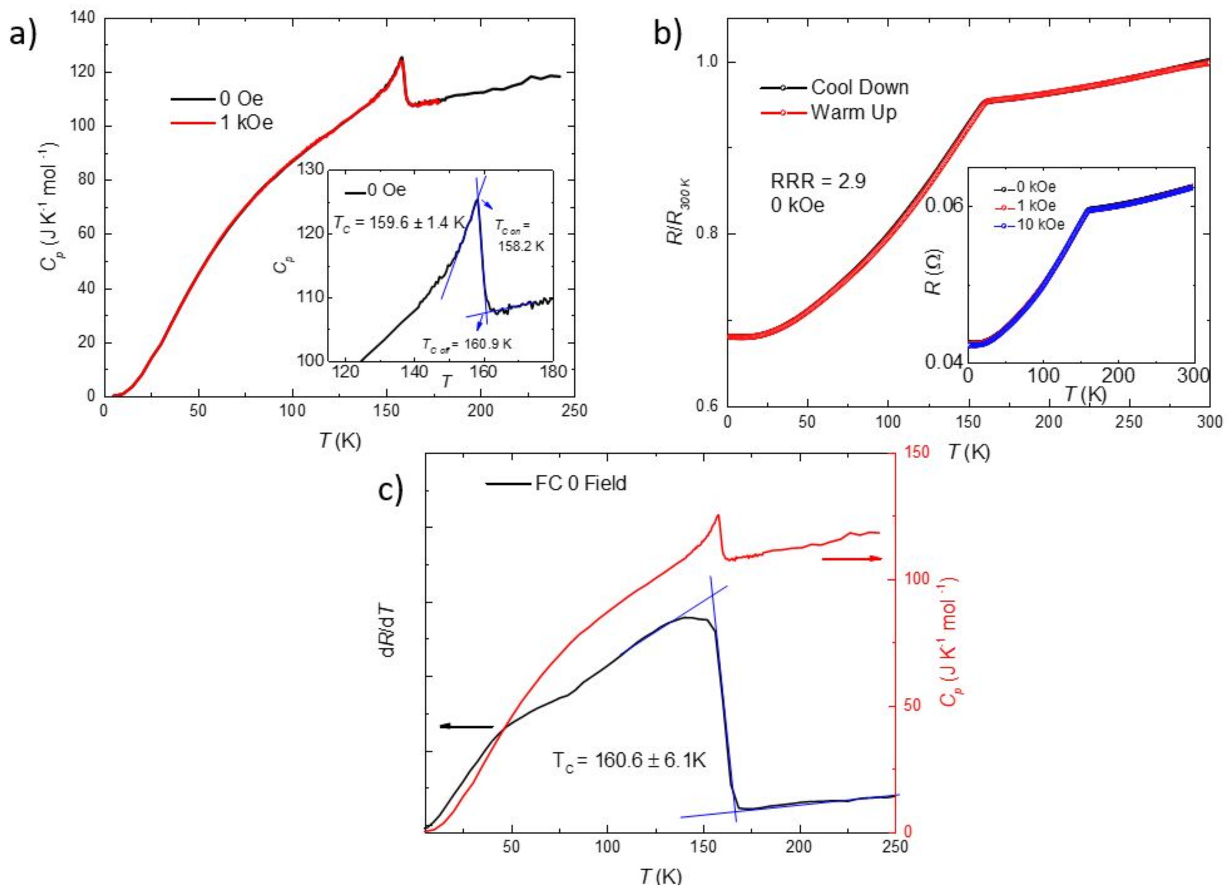

Fig. S4. Temperature-dependent specific heat. Fig. S4*a* shows the specific heat as a function of temperature under 0 (black) and 1 kOe (red). The (Insert) criteria used to determine T_C. **Fig. S4*b*** shows temperature-dependent resistance and insert gives the measurements under different fields. **Fig. S4*c*** presents the comparison between dR/dT and $C_p(T)$.

The ferromagnetic transition is determined by temperature-dependent specific heat and resistance measurements, as shown in **Fig. S4a** and **b**. Measurements are taken under 0 kOe and 1 kOe field perpendicular to the *c*-axis. These two measurements overlap, indicating negligible low-field influence on the specific heat capacity. A distinct second-order phase transition is observed, characterized by a well-defined peak in the specific heat curve, pinpointing the ferromagnetic transition temperature at 159.6 ± 1.4 K. In **Fig. S4*b***, the clear kink in temperature-dependent resistance is observed. The magnetic field, up to 10 kOe, was applied along *ab* directions, with the current flowing in the *c* direction. There is no clear different between zero and 10 kOe temperature-dependent resistance measurements. The transition temperature from resistance measurement is 160.6 ± 6 K, according to **Fig. S4*c***, which is close to the result of specific heat measurements with a larger transition width.

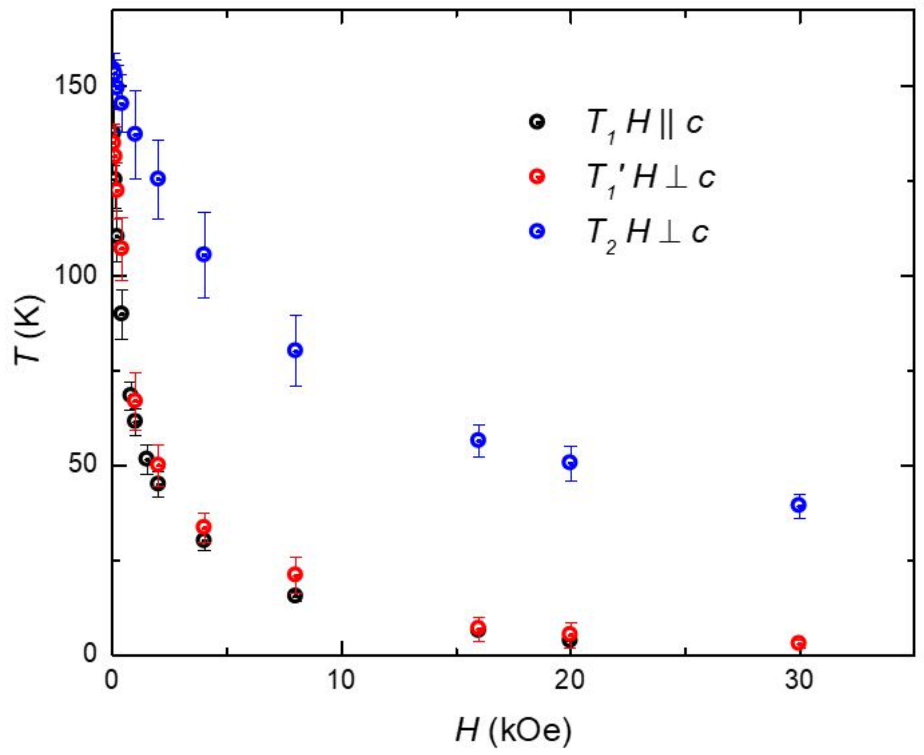

Fig. S5 diagram of T_1, T_1' and T_2. Fig. S5 shows jump temperature as a function of the magnetic field as the field parallel (black) and perpendicular (red and blue) to the crystallographic *c*-axis.

The temperatures associated with these jumps and kink-like features in zero-field-cooled-warming (ZFCW) magnetization are presented in **Fig. S5** as T_1, T_1', and T_2. These temperates are shown in **Fig. 2*a*** and **2*b***. An overlap between T_1 and T_1', the minimal change in magnetization of T_1', suggests that T_1 and T_1' correspond to identical magnetic features. The reason T_1' appears in the field perpendicular to the *c*-axis is not known. Considering the demagnetization field, T_1 and T_2 may change due to the shape of the different samples. This will not be discussed in this paper. Moreover, the analysis reveals a decrease in T_1 and T_2 values with increasing magnetic field intensity, further elucidating the magnetic characteristics of $SmCrGe_3$.

According to the CW fitting results, $SmCrGe_3$ has an effective moment is 1.54 μ_B compared with Sm^{3+} 1.74 μ_B. If we consider other rare-earth compounds. $LaCrGe_3$ is 2.5 μ_B compared

with Cr 3.7-4.8 μ_B. $CeCrGe_3$ is 3.18 μ_B compared with Ce^{3+} 2.54 μ_B. $PrCrGe_3$ is 3.8 μ_B, and $NdCrGe_3$ is 3.2 μ_B compared with Pr^{3+} 3.58 μ_B and Nd^{3+} 3.62 μ_B. Except for La and Ce, effective moments in other Ln113 have the CW fitting results closer to the Ln^{3+}. This may indicate that in these compounds, the 4*f* electrons play a more important role. As the only hard magnet in the Ln113, $SmCrGe_3$ has the largest coercivity, which is more than 100 times larger than other Ln113. All these make $SmCrGe_3$ interesting and worth studying.

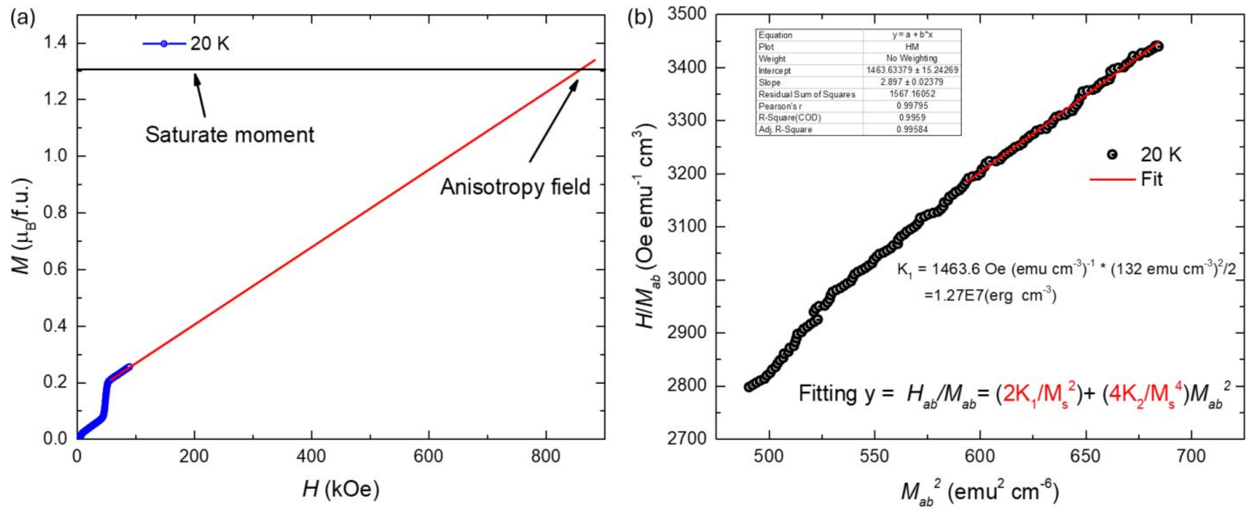

Fig. S6 ***a*** and ***b*** show the way to get the anisotropy field and anisotropy energy.

In **Fig. S6*a***, the saturation moment is estimated in the direction of the magnetic field parallel to the *c*-axis. In **Fig. S6*b***, the reason only choosing the high magnetic moment to fit is to make sure the magnetic domain disappears in this range. After getting K_1 and K_2, the energy is given by equation 1.
